# The protective effects of silymarin on ischemia-reperfusion injuries: A mechanistic review

**DOI:** 10.22038/ijbms.2019.34284.8147

**Published:** 2019-09

**Authors:** Vahid Akbari-Kordkheyli, Kazem Abbaszadeh-Goudarzi, Mohaddeseh Nejati-Laskokalayeh, Setareh Zarpou, Abbas Khonakdar-Tarsi

**Affiliations:** 1 Student Research Committee, Mazandaran University of Medical Sciences, Sari, Iran; 2 Cellular and Molecular Research Center, Sabzevar University of Medical Sciences, Sabzevar, Iran; 3 Department of Biochemistry-Biophysics and Genetics, Faculty of Medicine, Mazandaran University of Medical Sciences, Sari, Iran

**Keywords:** Inflammation, Ischemia, Oxidative stress, Reperfusion, Silymarin

## Abstract

Ischemia-reperfusion injuries (IRI) occur in different clinical conditions such as stroke, trauma, organ transplantation, and so on. Ischemia damages mainly arise from oxygen depletion in tissues. The lack of oxygen as the last acceptor of electron in the respiratory chain causes a decrease in ATP production and eventually leads to disruption of membrane transport, acidosis, cellular edema and membrane distortion of organelles, and cells. Reperfusion can intensify ischemic injuries by the infiltration of inflammatory cells and also oxygen and calcium overloading. Since the tissue antioxidant contents decreased due to increased generation of reactive oxygen species (ROS) during IRI, the application of antioxidants is considered an appropriate strategy to ameliorate IRI. Silymarin constitutes about 70–80% of silybum marianum dry extract and is known as a strong free radical scavenger with anti-inflammatory properties. In several studies, silibinin as a major component of Silymarin could provide protective effects in various tissue IRI by different mechanisms such as scavenging free radicals, decreasing inflammatory cytokines, inhibiting cellular death, and increasing the expression of antioxidant enzymes. To clarify functional mechanisms, the present article evaluates studies about silymarin effects in different tissues IRI.

## Introduction

Ischemia occurs during relative or complete obstruction of tissue blood circulation. Ischemia damages mainly arise from oxygen depletion in the tissue. In pre-acute phase, the lack of oxygen as the last acceptor of electron in the respiratory chain causes a decrease in ATP production and eventually leads to the disruption of membrane transport,‏ acidosis, cellular edema and membrane distortion of organelles, and cells (1, 2). 

Blood flow restoration into the ischemic tissues known as reperfusion is a vital process to compensate oxygen deficiency and eliminate any cytotoxic metabolites accumulated during ischemia. It should be noted that it can intensify ischemic injuries, about 12–24 hr after reperfusion, named IRI (3, 4). During reperfusion, the high influx of blood into the ischemic tissues results in the infiltration of inflammatory cells, and oxygen and calcium overloading which can increase the generation of ROS. In addition to local tissues, IRI triggers a systemic inflammatory response and multiple organ dysfunctions via metabolite distribution, such as inflammatory cytokines (5, 6).

IIRIs occur during different clinical conditions such as vascular obstruction, myocardial infarction, thrombolytic treatment, orthopedic surgeries, hemorrhagic shock, cardiopulmonary bypass, revascularization, and organ transplantation which is considered the main one (7-10). The clinical manifestations of IRI include myocardial hibernation/stunning, cerebral dysfunction, destruction of the gastrointestinal barrier, systemic inflammatory responses, and multiple organ dysfunctions (11-13).

The clinical factors affecting the intensity of IRI include the duration and severity of ischemia, reperfusion rate, organ health status, and age of the affected person (14). There are many contemporary treatment strategies for IRI whose supporting effects have been tested in experimental studies and clinical trials, e.g., the application of anti-inflammatory drugs (dexamethasone, prednisone and tacrolimus), inhibitors of broad spectrum of serine proteases (aprotinin), selective inhibitor of Na+/H+-exchange (cariporide), anti-apoptotic agent (Bax inhibitor-1), anti-ischemic component (trimetazidine), antioxidants (SOD, CAT, N-acetyl cysteine, vitamin E and D, melatonin), ischemic preconditioning induction, and controlled reperfusion (15-20). Despite different studies, IRI is still an unresolved problem in different clinical conditions. Many researchers have shown that the severity of the damage depends on the rate of antioxidant exposure to the tissues. Since the tissue antioxidant contents decreased due to a large amount of ROS during IRI, the application of antioxidant agents is considered an appropriate strategy to ameliorate IRI (21-24).

Silymarin makes up about 70–80% of *Silybum marianum*; it is known as a strong free radical scavenger with anti-inflammatory and anticancer properties (25, 26). Also, several investigations demonstrated that silymarin and its main component silibinin act against different biological (bacterial toxins and mycotoxins) and chemical (pesticides, metals, fluoride, cardiotoxic, and hepatotoxic) poisons (27-30). More than 400 articles have been published about the beneficial effects of silymarin and its components in the last few years. The article’s focus is to review the functional mechanisms of silymarin and silibinin on IRI.

**Pharmacology of silymarin**

**Figure 1:**
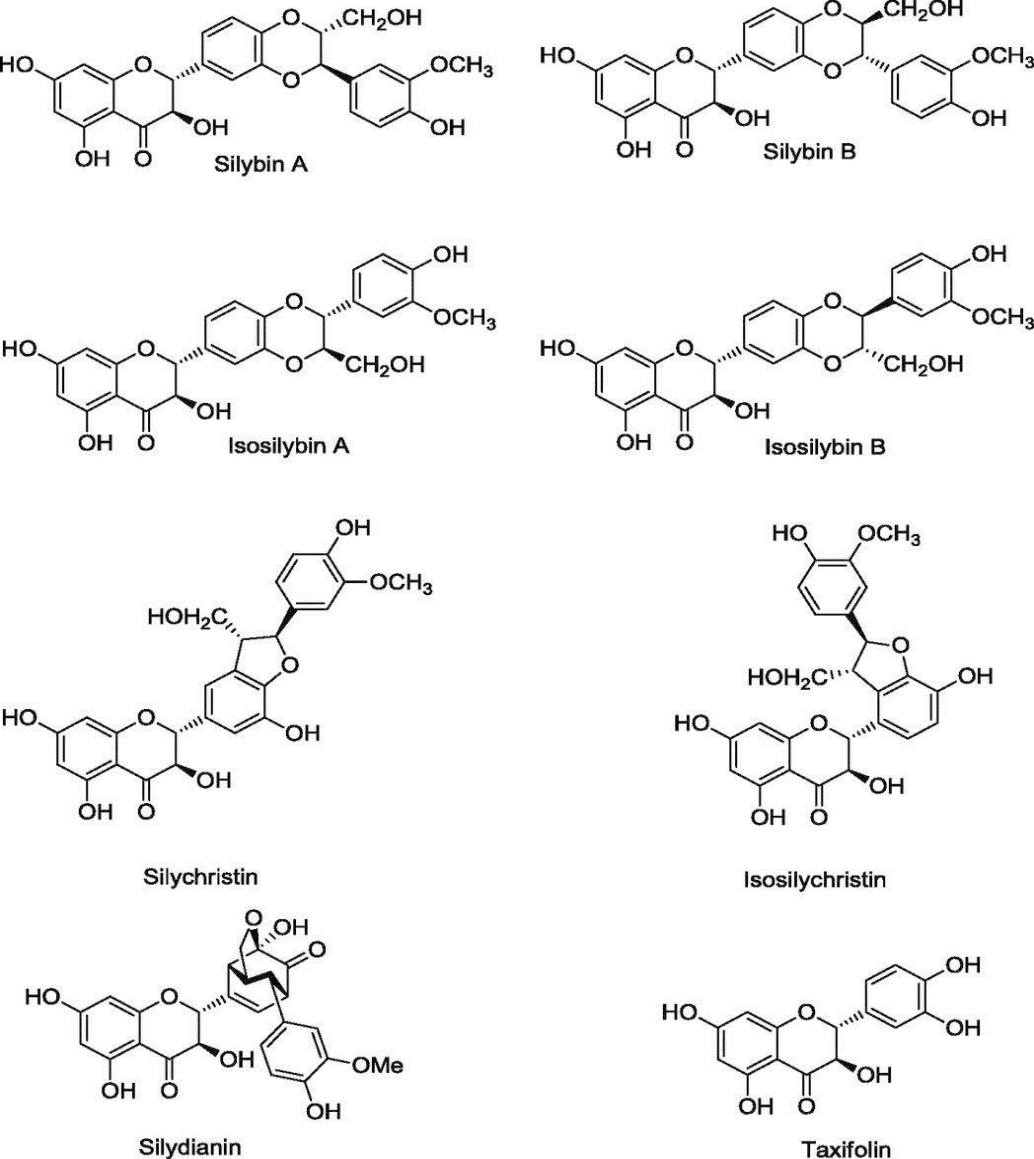
The structure of silymarin components

Silymarin consists of seven flavonolignans including silybin A and B, isosilibin A and B, silychristin, isosilychristin, silydianin and two flavonoid compounds, taxifolin, and quercetin (Figure 1). Silibinin or mixture of silybin A and B constitutes about 60–80% of silymarin components and its main effective ingredient (25). After a little intestinal absorption (20–30%) of silymarin (silibinin), about 70–80% is conjugated with glucuronide and rapidly excreted through the bile system. Furthermore, the low blood concentration of silymarin explains its less adverse effect. In some liver and kidney disorders, the unconjugated form increased in circulation, which is biologically active (31, 32). Based on the non-ionizable structure of silymarin, it has low solubility in aqueous solutions, about 0.5 g/l. Organic solvent solubility is about 0.1, 10, and 20 g/l in ethanol, dimethyl sulfoxide (DMSO), and dimethylformamide, respectively. In this regard, the application of conjugated forms such as silibinin phosphatidylcholine (siliphos) and silibinin dihydrogen succinate disodium (Legalon) are in preference due to their high solubility in aqueous solution. The serum half-lives of silibinin are about two and three hours for free and protein-conjugated forms, respectively (32-34). 

The supportive effects of silymarin on tissue IRI are well-documented. Silymarin can improve total antioxidant capacity by scavenging free radicals and elevating antioxidant gene expression. It is able to suppress inflammatory response by inhibiting the activation of NF-κB and cyclooxygenase-2 (COX2) (35, 36). Silymarin’s antiapoptotic properties are exerted by preventing the release of cytochrome (Cyt c) and inhibiting the activation of caspase (37, 38). We found that inflammatory response, oxidative stress, and cell death are the major causes of IRI. In the present study, we evaluate the effects of silymarin on these injuries. 

**Figure 2:**
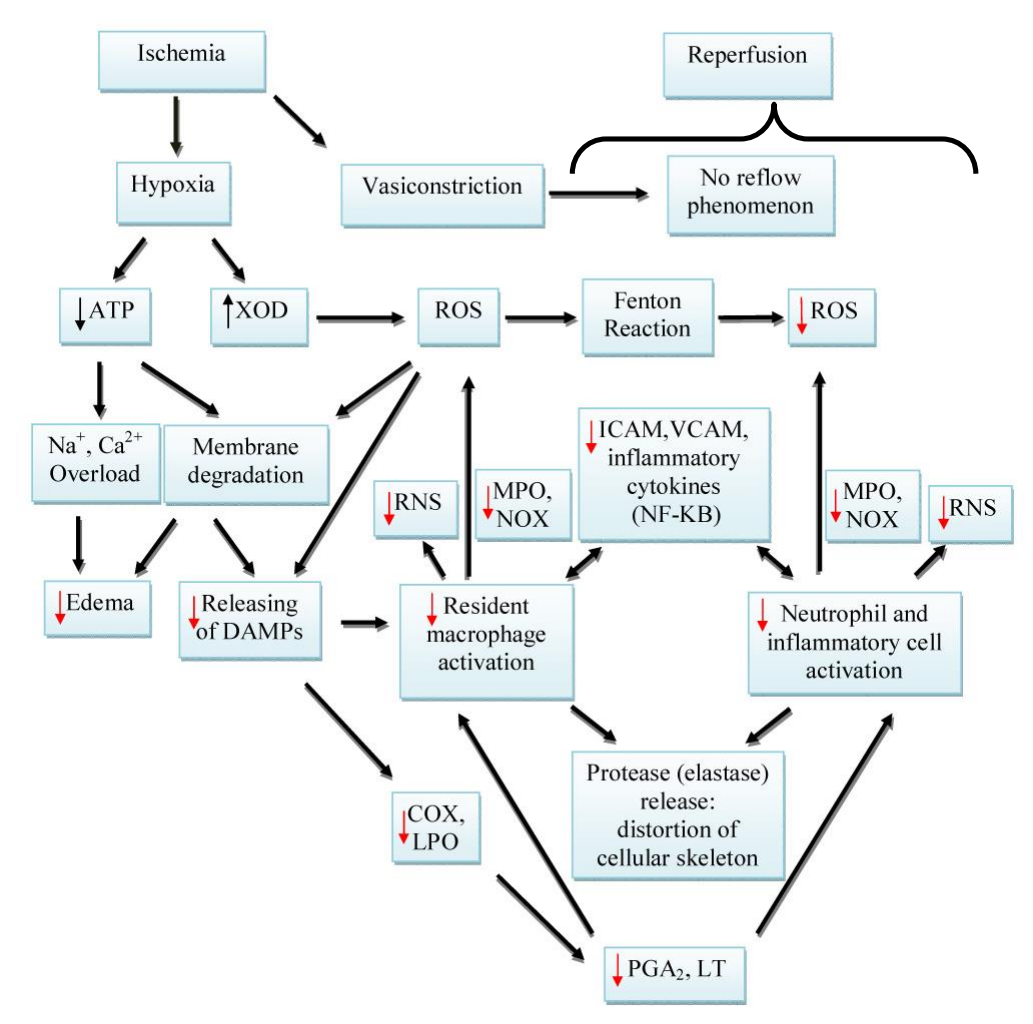
Inflammation process during ischemia-reperfusion. ↓ Decreased by silymarin/silibinin. COX: Cyclooxygenase; DAMPs: Damage-associated molecular pattern; ICAM: Intercellular adhesion molecules; LPO: Lipoxygenase; LT: Leukotrienes; MPO: Myeloperoxidase; NF-κB: Nuclear factor-Κb; NOX: NADPH oxidase; PGA2: Prostaglandin A2; RNS: Reactive nitrogen species; ROS: Reactive oxygen species; VCAM: Vascular cell adhesion molecule

**Figure 3:**
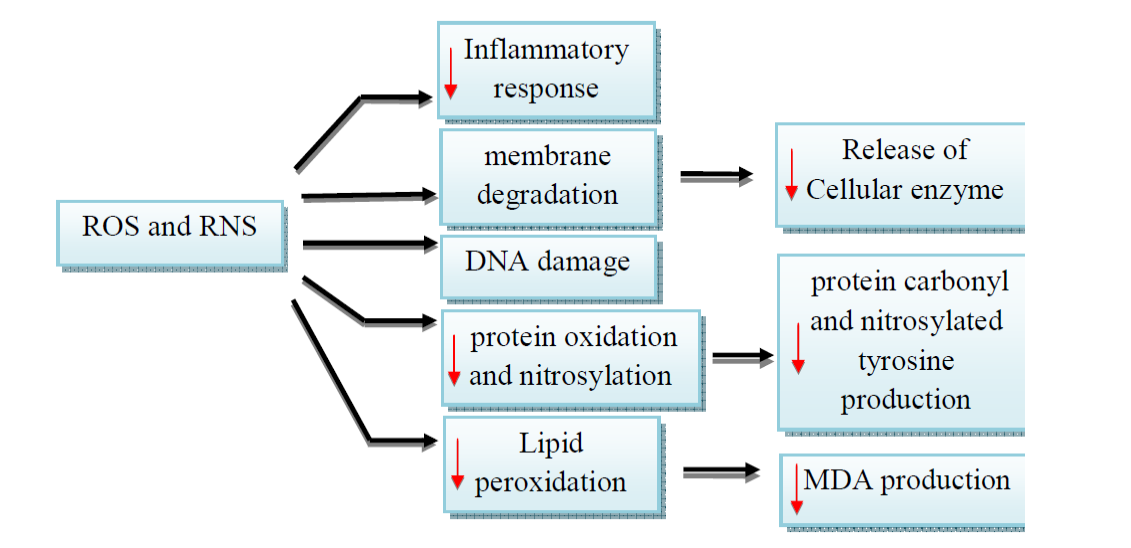
ROS and RNS-associated injuries during ischemia-reperfusion. ↓ Decreased by silymarin/silibinin

**Figure 4:**
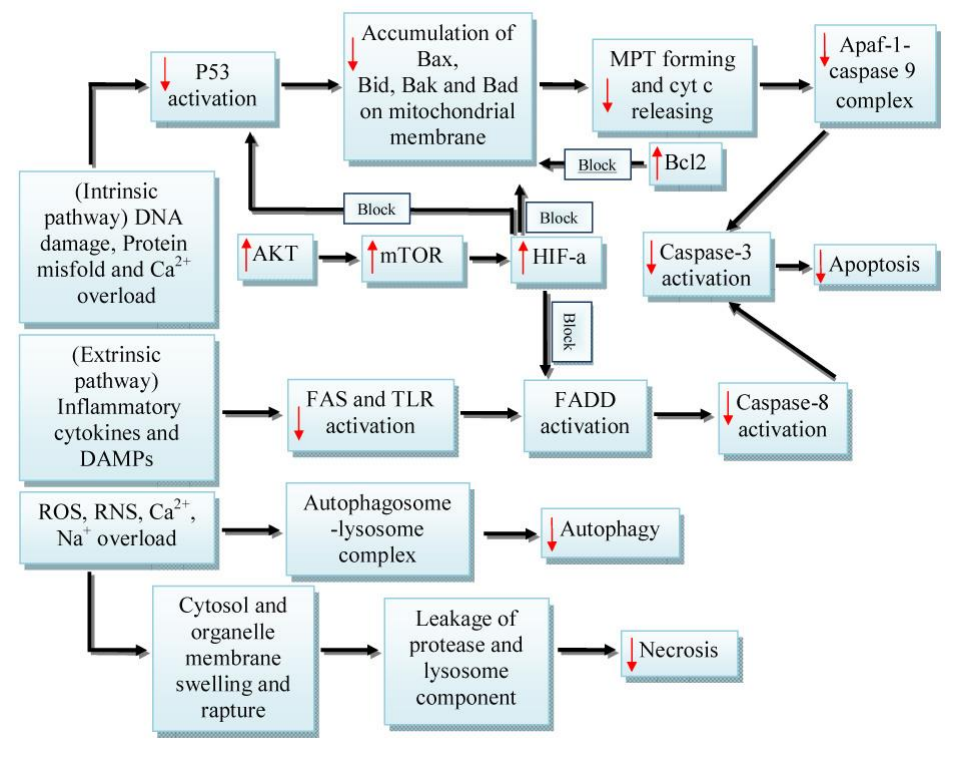
Cell death processes during ischemia-reperfusion. ↓ Decreased by silymarin/silibinin, ↑ Stimulated by silymarin/silibinin. Apaf-1: Apoptotic protease activating factor 1; Cyt C: Cytochrome C; FADD: Fas-associated protein with death domain; HIF-a: Hypoxia-inducible factor 1-alpha; mTOR: Mammalian target of rapamycin; MPT: Mitochondrial permeability transition; TLR: Toll-like receptor

**Table 1:** Silymarin/silibinin effects on tissue ischemia-reperfusion

**Tissue I/R**	**Effects**	**Ref**
Kidney	Decreased tubular vacuolation and dilatation, hyaline casts, hyperemia, cellular edema, and serum creatinine	(46, 65, 72, 97)
Stomach	Diminish mean ulcer index	(50)
liver	Improved ATP level, mitochondrial function, and respiratory chain parameters. Reduced AST, ALT, GGT, total bilirubin, vaculation, edema, hyperemia, hydroxyproline, and sinusoidal congestion, and increased glycogen phosphorylase activities	(55, 69, 70, 98, 99)
Multiple organs	Prevent intestinal edema, loss of intracellular border in the liver, alveolar congestion, and hemorrhage	(77)
Cerebral	Relieve infraction size, memory impairment, water content, and neurobehavioral alteration	(52, 53, 92, 100)
Coronary artery occlusion	Ameliorate blood pressure, ventricular hypertrophy, and heart arrhythmia, and abrogate LDH and CK	(57, 101)

IR: Ischemia-reperfusion

**Ischemia-reperfusion injuries and silymarin helpful effects**

***Inflammatory response during IRI***

Inflammation is one of the main mechanisms of IRI, especially during the lateral phase. In ischemic tissues, macrophages are activated by the release of damage-associated molecular patterns (DAMPs) from injured cells. The macrophages secrete inflammatory cytokines, mostly IL-1β, TNF-α, and IL-6 leading to other inflammatory cells especially neutrophils. The infiltration of inflammatory cells triggers the production of ROS and reactive nitrogen species (RNS) by pathways such as myeloperoxidase (MPO) and inducible nitric oxide synthase (iNOS). According to Figure 2, inflammatory cells can produce proteases such as elastase that distort cellular skeleton (39-42). Microvascular aggregation of inflammatory cells reduces blood fluidity, which results in the no-reflow phenomenon during reperfusion (43). It should be noted that vascular endothelium, which is directly exposed to blood’s mechanical force, responds earlier to circulation abnormalities. Microvascular contraction is another cause of the no-reflow phenomenon that begins in ischemia and continues during reperfusion (44, 45).

any investigations have proven the anti-inflammatory effects of silymarin during ischemia-reperfusion (IR). Investigation clarified that pretreating rat kidney tissues exposed to 45 min ischemia followed by 24 hr reperfusion with silymarin (100 mg/kg, iv) could decrease the levels of IL-6, IL-1β, TNF-α, MPO activity (an indicator of inflammatory cell infiltration), and CD65 gene expression (expressed in high levels in monocyte/macrophage) (46). 

Silymarin administration during kidney IRI, reduced urinary kidney injury molecule 1 (KIM-1) (47) and neutrophil gelatinase-associated lipocalin (48) and increased inhibitor of NF-κB (I-κB) (49). A study examined the gastroprotective effects of silymarin during IRI; ischemia was induced by occlusion of the celiac artery for 30 min, and reperfusion lasted for 60 min. The results showed that the number of neutrophils in the gastric mucosa and circulation and MPO activities were diminished by silymarin (50–100 mg/kg, IV), but not as well as dexamethasone (50). Anti-inflammatory properties of silymarin on lung IRI was identified. Silymarin (250 mg/kg, IV) to be taken each day for seven days before surgery, could decline serum levels of IL-6, IL-1β, TNF-α, NF-κB, hypoxia-inducible factor-α (HIF-α) and iNOS protein expression by lung tissue (51). 

A study demonstrated that the brain tissue levels of COX_2_, intracellular adhesion molecule-1 (ICAM-1), P_65_ NF-κB, TNF-α, IL-1β, iNOS, and I-κB degradation were suppressed by silymarin (1-10 mg/kg, iv) after cerebral IR (1 hr ischemia and 24 hr reperfusion) (52). Other studies on cerebral IR indicated that inflammatory cell infiltration, leukotriene synthesis, phagocytosis, and edema were prevented by silymarin (53, 54). Younis *et al*. tested the efficacy of silymarin in insulin-resistant rats; the liver of rats underwent 30 min ischemia followed by 1 hr reperfusion. Treatment by silymarin (100 mg/kg, IV), 15 min before reperfusion decreased the serum levels of TNF-α and nitrite, while the levels of IL-10 (an anti-inflammatory cytokine) were increased (55). 

Anti-inflammatory effects of silymarin are mostly due to inhibiting the nuclear translocation/activation of NF-κB that resulted in reducing inflammatory cytokines. These events led to preventing the aggregation of inflammatory cells, which was followed by the reduction of iNOS and MPO activities (56, 57). It has been reported that silymarin is able to suppress 5-lipooxygenase and COX activities that lead to inhibiting leukotriene and prostaglandin production (58, 59).

***Oxidative stress***

Oxidative stress is considered the main mechanism of IRI. Following the destruction of the mitochondrial membrane during ischemia, dangerous components such as Cyt c and xanthine dehydrogenase are released (60). Under oxidative stress conditions, xanthine dehydrogenase is converted to xanthine oxidase, which is the main source of intracellular ROS during IR. The enzyme produces H_2_O_2_, which could be converted to OH^-^ as a result of an ion entrance into the cell during reperfusion and undergoes the Fenton and Haber-Weiss reactions. A large amount of ROS and RNS such as HCLO, NH_2_Cl, and ONOO^-^ is generated by infiltrating inflammatory cells via MPO, NADPH oxidase (NOX_2_) and iNOS pathways. In this condition, the body’s antioxidants such as catalase, glutathione peroxidase (GPX) and superoxide dismutase (SOD) neutralize the reactive components. Because of the large amount of free radicals that exceeded the body’s antioxidant capacity, this resulted in peroxidation of proteins, lipids, and DNA (Figure 3) (61-64). 

Many studies have been done on silymarin/silibinin antioxidant effects during tissue IR. Flavonoids are strong free radical scavenger due to a multi-phenolic structure. Due to large amount of lipids deposit, high oxygen consumption, and low levels of antioxidants, the brain is very sensitive to ROS. Therefore, suppressing oxidative stress is in preference to decrease brain IRI (60). In the investigation of Rui *et al*. the cerebral effects of silibinin in rats have been evaluated; IR was induced by the obstruction of the carotid artery for 30 min followed by 2 hr reperfusion. Treatment with silibinin could diminish malondialdehyde (MDA) as a lipid peroxidation yield and improve SOD activity in brain tissue. The results revealed that the efficacy of the drug was better in the dosage of 400 than 100 and 200 mg/kg (54). In some other studies, the antioxidant effect of silymarin on cerebral IR was also reported via abrogating nitric oxide (NO) and nitrosylated tyrosine (NO-Tyr), while improving CAT (65), GPX, and glutathione reductase (GR) activities (66). Ergün and coworkers investigated the impacts of silibinin (50 mg/kg, IP) prior to reperfusion on skeletal muscle IR injury. Taken to gather, silibinin could not meaningfully affect the SOD and CAT activities and MDA level (67). 

In the other study, researchers utilized silymarin and carvacrol in liver IRI; it was identified that these drugs could decrease MDA, while increasing glutathione-SH levels and improving CAT activity (68, 69). An experiment showed that silibinin increases liver GSH levels and CAT activity during liver IRI (70). Cetinkunar *et al*. also tested the impact of silymarin on hepatectomy induced injuries. After clamping the left branch of the portal triad, two lobes of liver were harvested, treatments with silymarin (200 mg/kg) to be taken each day for six days decreased liver MDA, but no changes were seen in SOD and GSH levels (71). 

A study examined the effects of different doses of silymarin on rat kidneys. Results demonstrated that SOD and CAT were improved, while MDA was diminished in the kidneys. Silymarin has been used in different doses; the optimum dose of silymarin (200 mg/kg) had better efficacy than the other doses (72). Based on experimental research performed, rat kidney was exposed to 45 min ischemia followed by 24 hr reperfusion; the results illustrated that silymarin could increase the serum levels of SOD, GPX, and total antioxidant capacity (73) during IRI, but no effect on the levels of SOD and GPX in renal tissue. Silymarin could decrease the level of MDA, while increasing TAC and antioxidant enzyme activities via scavenging free radicals and elevating antioxidant gene expression (74) in different organs that experienced IRI including mesenteric (75), myocardium (57), kidney (46, 49), corporal (76), and supraceliac (77).

***Cell death***

During IRI, irreversible lesions conduct a group of cells to death. As a result of changes in membrane transport, an influx of Ca^2+^ and ROS creation, proapoptotic proteins are activated that can trigger mitochondrial permeability transition pore (MPTP) formation (78). The release of mitochondria components such as Cyt c can stimulate caspase-3 and -9 activities that lead to apoptosis and necrosis (79). Some studies indicated that glycolysis-induced acidosis during ischemia prevents MPTP formation. However in reperfusion, after normalizing of pH, MPTP can exist. Therefore, cell death usually occurs during reperfusion (80).

 Apoptosis can happen with intracellular pathways such as DNA injuries, p53 activation, excess glycolysis, Cyt c excretion, and extracellular pathways via death receptors. It is proven that in intracellular pathways, B cell leukemia/lymphoma 2 (Bcl2) family proteins are the most important regulators. The Bcl2 family proteins consist of proapoptotic members (Bax, Bak, Bad, and Bid) and antiapoptotic members (Bcl-2, Bcl-Xl, and Bcl-W). In extracellular pathways, inflammatory cytokines such as tumor necrosis factor α (TNF-α) and Fas ligand (FasL) have the main role in apoptosis. The receptor activation can lead to caspase-8 activity that finally stimulates caspase-3 activation (Figure 4). During MPTP formation, ATP production is stopped, which resulted in the inhibition of caspase activation, plasma membrane degradation and eventually necrotic cell death known as necroptosis (81-83). Also recently the role of microRNAs in regulation of mitochondrial apoptotic pathways has been determined during I/R. For example miR-1 and miR-15 induce proapoptotic factors by targeting Bcl-2 and Arl2, respectively, and miR-21 and miR-22 increase anti-apoptotic proteins by targeting AP-1 and P53, respectively during I/R (84, 85).

Silymarin’s beneficial effects on cell death during IR are well established; a study showed that administration of silibinin in conjugated forms with hydroxypropyl-β-cyclodextrin during hepatic IR could reduce the protein expression of apoptosis such as Fas ligand (FasL), high mobility group box-1 protein (HMGB1) and lymphocyte common antigen (LCA) as a CD45 (86). HMGB_1 _is released by apoptotic and necrotic cells after stimulating the toll-like receptors (TLRs) by damage-associated molecular patterns (DAMPs) considered a cell death marker (87). Death receptors such as Fas and its ligand are stimulated during IR (88). Pre-treatment with silymarin could abrogate caspase-3 and-9 levels after pulmonary IR (51). 

Cetinkunar *et al*. proved the anti-necrotic properties of silymarin after partial liver hepatectomy (71). Moreover, a study tested silibinin effects on kidney damages induced by hepatic IR. It is identified that M30 as an apoptotic biomarker decreased by silibinin (89). By increasing Bcl_2 _as an antiapoptotic protein and decreasing Bax as a proapoptotic protein in kidney IR, silymarin decreased apoptotic cells (46). Bax is a protein that activates the cleavage of caspase-3 and induces apoptosis. It should be noted that the ratio of Bax/Bcl_2_ determines whether cells experience apoptosis or survival (90). 

In an experiment, silymarin reduced signal transducer and activator of transcription-1 (STAT-1), p38 mitogen-activated protein kinases (MAPKs) and caspase-3 and 9 during cerebral IR (91). Research showed that silymarin reduced the expression and activities of the apoptotic protease activating factor-1 (Apaf-1), P_53_, and caspase-3 and 9 in rat brain insulted by IR (92). P_53_ activation triggers the release of Cyt c and is able to activate caspase-3 and 9 via apaf-1 (93). In a study performed on ischemic stroke, silymarin treatment (100 mg/kg) could inhibit the expression of Bax and NF-κb proteins, while inducing Bcl_2_, HIF-α, pAKT, and pmTOR levels in brain tissue (94). It should be noted that phosphorylated AKT (pAKT) and phosphorylated mTOR (pmTOR) can protect neurons from death through anti-apoptotic and anti-inflammatory actions by inducing HIF-α (95). 

The protective role of silymarin on cerebral IR is now well documented. In a study, a rat’s brain was exposed to 20 min ischemia followed by seven days reperfusion. One group of rats received silymarin (250 mg/kg), hematoxylin and eosin (H&E) staining; results revealed that the treatment had no effect on the cellular density of the hippocampus region during IR. Fluoro-Jade B (FJB) staining demonstrated that cell death was less in the treated group. Furthermore, terminal deoxynucleotidyl transferase-mediated dUTP nick end-labeling staining showed that silymarin was able to decrease apoptotic cells in brain tissue during IR (96). 

It seems that silymarin/silibinin can prevent intra- and extra-cellular pathways of apoptosis through stabilizing cell and mitochondria membranes, while decreasing inflammatory cytokines. Silymarin/silibinin can induce the expression of anti-apoptotic proteins such as Bcl_2 _and HIF-α while inhibiting the expression of proapoptotic proteins such as caspase families and Bax by activating signal transduction pathways.

It should be mentioned that the mechanisms of IRI are various, complex, and difficult to categorize. It is also proven that silymarin can affect multiple pathophysiological pathways (25). In this regard, various beneficial impacts of silymarin are reported during IR; some of the impacts are summarized in Table 1.

**Conclusion and perspectives IR**

The pathogenesis of IRI is complex and multifactorial; it involves ATP depletion, changes in membrane transportation, cellular edema, glycolysis-associated acidosis, microcirculation defects, inflammation, oxidative stress, and cell apoptosis. In different pathological status, the protective effects of silymarin make it attractive to utilize in IRI. Based on studies, the main action mechanisms of silymarin/silibinin are scavenging free radicals, increasing antioxidant capacity, preventing an excess inflammatory response, and inhibiting different types of cell death. These events lead to improved organ function after reperfusion. As mentioned, microcirculation impairments and miRNAs dysregulation may have a key role in IRI. In this regard, the authors strongly recommend investigating the efficacy of silymarin/silibinin in microcirculation related signal transduction and miRNAs pathways during tissue IR. 
